# Role and intervention of PAD4 in NETs in acute respiratory distress syndrome

**DOI:** 10.1186/s12931-024-02676-7

**Published:** 2024-01-30

**Authors:** Xiaojie Liu, Tianjun Li, Huailong Chen, Li Yuan, Hushan Ao

**Affiliations:** 1 Department of Anesthesiology, The Affiliated Hospital of Qingdao University, No. 16, Jiangsu Road, Qingdao, Shandong China; 2 Department of Oncology, The Affiliated Hospital of Qingdao University, No. 59, Haier Road, Qingdao, Shandong China; 3 Department of Anestheiology, The Qingdao Eighth People’s Hospital, No. 210 Jinshui Road, Licang District, Qingdao City, Shandong China; 4 Department of Anesthesiology, Fuwai Hospital, Chinese Academy of Medical Sciences and Peking Union Medical College, No.167 North Lishi Road, Xicheng District, Beijing, China

**Keywords:** ARDS, Sepsis, NETs, PAD4

## Abstract

**Background:**

Sepsis is life-threatening organ dysfunction caused by a dysregulated host response to infection. Acute respiratory distress syndrome (ARDS) is a common sepsis-associated injury that can increase postoperative mortality but the mechanism is still unclear.

**Main text:**

The role of neutrophils in the pathophysiology of sepsis was deeply challenged after the discovery of NETosis, a process resulting in neutrophil extracellular traps (NETs) release. NETs can support thrombin generation and the concept of immunothrombosis has emerged as a new innate response to infection. Immunothrombosis leads to thrombosis in microvessels and supports immune cells together with specific thrombus-related molecules. ARDS is a common sepsis-associated organ injury. Immunothrombosis participates in thrombosis in pulmonary capillaries. Intervention regarding immunothrombosis in ARDS is a key scientific problem. PAD4 is the key enzyme regulating the NET skeleton protein histone H3 to citrulline histone to form NETs in immune thrombosis. This review summarizes NETosis and immunohaemostasis, ARDS and therapeutic opportunities targeting PAD4 via PAD4 inhibitors and lncRNAs potentially, providing future therapies.

**Conclusions:**

We identified and summarized the fundamental definition of ARDS and the concept of immune thrombosis and its composition. NETs activation has become particularly relevant in the formation of immune thrombosis. The taskforce highlighted the intervention targets of PAD4, including noncoding RNAs, potentially providing future therapeutic targets to confront the high postoperative mortality of ARDS.

## Introduction

The proportion of acute respiratory distress syndrome (ARDS) mortality has been challenging and was estimated for sepsis induced at 27–37% [1]. Sepsis and multiple organ failure are more common causes of death than respiratory failure [2, 3]. Okazaki et al. investigated the effect of the potential interaction between sepsis and ARDS. The mortality in the ARDS group was higher than that in the non-ARDS group in the nonsepsis group. The mortality in ARDS and non-ARDS subgroups was similar to that in the sepsis group [4]. Lung is the first easily damaged sepsis-induced organ [5]. The signaling pathways related to alveolar injury and repair in sepsis-induced ARDS were summarized, including the NF-κB, JAK2/STAT3, mitogen-activated protein kinase (MAPK), mTOR, and Notch signaling pathways [6]. Currently, treatment of ARDS and sepsis has consisted mostly of supportive care, as targeted therapies have largely been unsuccessful. The definition of Berlin-defined ARDS: acute lung injury (less than one week) with bilateral inflammatory œdema and PaO2/FiO2 ratio less than 300 mmHg (mild), less than 200 mmHg (moderate) or less than 100 mmHg (severe) [7, 8]. ARDS can be associated with cardiovascular and cerebrovascular events caused by thrombosis, such as venous thromboembolism, myocardial infarction and stroke, which are the main complications, most of which are caused by the destruction of host defense function. COVID-19 has infected millions of people worldwide. The acute respiratory infection by SARS-CoV-2 resulted in pneumonia with hypoxaemia and bilateral images on CT-scan [9]. SARS-CoV-2 infects the host using the angiotensin converting enzyme 2 (ACE2) receptor, which is expressed in several organs, including the lung, heart, kidney, and intestine. ACE2 receptors are also expressed by endothelial cells. Study illustrated that SARS-CoV-2 infection facilitates the induction of endotheliitis in several organs as a direct consequence of viral involvement and of the host inflammatory response. This strategy could be particularly relevant for vulnerable patients with pre-existing endothelial dysfunction, which is associated with male sex, smoking, hypertension, diabetes, obesity, and cardiovascular disease, all of which are associated with adverse outcomes in COVID-19 [10]. Endothelial cell injury leads to inflammation and coagulation dysfunction, resulting in disorders [11].

The major features in the lungs of COVID-19 patients comprised primary inflammatory thrombosis associated with diffuse alveolar damage. The lungs had pronounced circulatory changes with inflammation-dependent intravascular blood clotting, whereas heart, brain, and kidneys had predominantly degenerative changes that were distinct from the lung pathology [12]. Findings identified a specific radiologic pattern of coronavirus disease 2019 (COVID-19) pneumonia with a unique spatial distribution of pulmonary vascular thrombosis (PVT) overlapping areas of ground-glass opacities [13]. Moreover, the thrombi evidenced in the pulmonary vasculature are “in-situ thrombosis”. This rate of pulmonary embolus is higher than usually encountered in critically ill patients without COVID-19 infection or in emergency department patients [14]. Why thrombotic events were increased in ARDS patients need further study.

## NETosis and immunohaemostasis

**Fig. 1:**
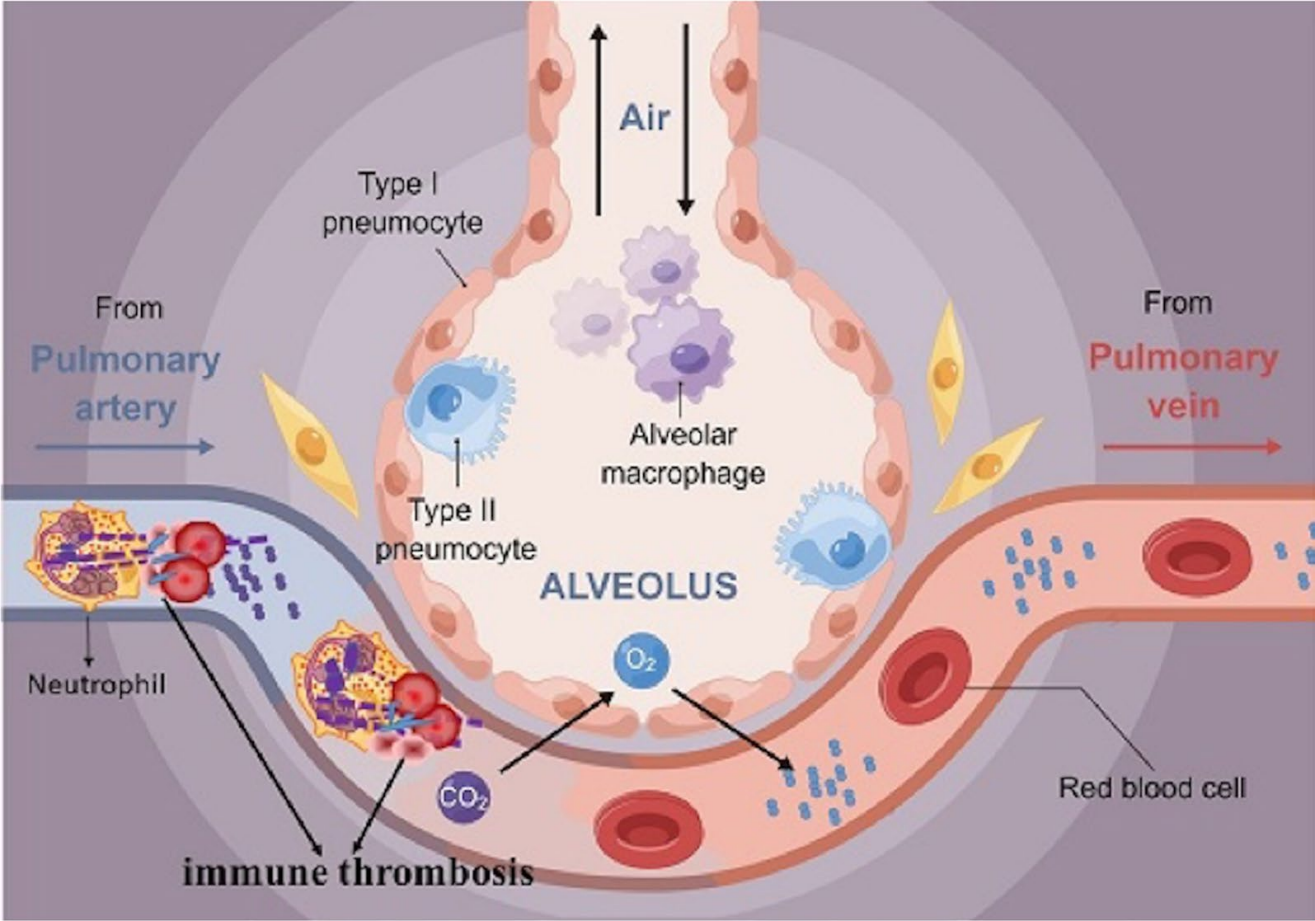
The figure shows the capillaries in the alveoli. Immunothrombosis is composed of polymorphonuclear neutrophil, platelets and erythrocytes. Illustrating the narrowing the capillaries, affecting the exchange of blood and gas in the alveoli

In 2013, Engelmann et al. first defined "immune thrombosis" [15], which is an innate immune response that leads to thrombosis in microvessels and supports immune cells together with specific thrombus-related molecules (Fig. 1). Immune thrombi can promote the recognition, trapping and disposal of pathogens and protect the integrity of the host; under normal circumstances, it will not cause significant damage. If the reaction lasts for a long time, it may lead to a cascade of blood clots, blocking tiny blood vessels and causing organ damage or even death.

Neutrophil extracellular traps (NETs), first reported by Brinkmann et al. in Science in 2004, are a kind of network structure that neutrophils release into the extracellular space after activation. DNA is the skeleton of NETs and is composed of histones, myeloperoxidase (MPO), neutrophil elastase, cathepsin G and other bactericidal proteins [16]. Tissue factor contribute to vessel-mediated thrombin generation and activation of the clotting pathway mediated by anionic surface-related contact proteins, thus leading to the formation of thrombosis in the vascular system [17].

The core component of NETs is polymorphonuclear neutrophils (PMNs). Uncontrollable activation of PMNs releases many oxygen free radicals and a variety of proteolytic enzymes, damaging tissue cells and vascular endothelial cells. However, these cannot only easily used clinically but also increase costs. Drug therapy is widely used in clinical work due to its convenient use and low cost. Previous research reviewed the protective effects of drugs in various targets after cardiothoracic surgery and discussed the mechanisms. However, its application is limited due to its single target and downstream action site [18].

Previous studies have shown that β2 integrin of neutrophils plays a major role in bridging inflammation and the coagulation response, which cannot play a key role due to its single site [19]. Recent data suggested that human neutrophils could survive up to 5.4 days in the circulation [20]. The high number of neutrophils emerging from the bone marrow on a daily basis 10 [9] cells/kg of body weight per day) is balanced with their short half-life in circulation (up to approximately 12 h) [21]. In contrast, the abundance of neutrophils in humans, representing up to 70% of circulating leukocytes, is not reflected in mice. The percentage of neutrophils varies from 10 to 25% of white blood cells.

CRP also promotes mononuclear endothelial cell interactions and both plasminogen activator inhibitor-1 (PAI-1) and tissue factor (TF) expression. Our previous study found that there were many factors affecting the coagulation response after surgery in elective coronary artery bypass grafting [22]. Preoperative C-reactive protein (CRP) is significantly correlated with activated partial thromboplastin time (aPTT) in the endogenous coagulation pathway, which is speculated to be involved in the endogenous coagulation system [23]. However, whether CRP is involved in the activation of endogenous clotting pathways by surface proteins requires further investigation.

The effect of heparin on mortality in sepsis is still debated. A previous study indicated that heparin may be associated with decreased mortality in patients with sepsis, septic shock, and disseminated intravascular coagulation. The overall impact remains uncertain [24]. Increased major bleeding with heparin administration and safety outcomes require further study. Large randomized trials are needed to evaluate the efficacy and safety of heparin in patients with sepsis, severe sepsis, and septic shock more carefully. Further studies illustrated that in addition to routine treatment, LMWH can reduce the incidence of multiple organ dysfunction syndrome (MODS) and 28-day mortality rate and improve the prognosis of adult patients with sepsis by improving coagulation function and reducing inflammatory reactions and the risk of bleeding [25]. Continuous anticoagulation therapy was used with low-dose heparin and low-molecular-weight heparin to improve the prognosis and reduce the mortality of patients with ARDS.

The 28-day fatality rate in the experimental group was significantly lower than that in the control group. The 7-day SaO_2_ was significantly increased. The APACHE II score, the lung injury score and the respiratory rate in the experimental group were significantly lower than those in the control group after 7 days of treatment. The length of ICU and mechanical ventilation in the experimental group were lower than those in the control group. After 7 days of treatment, the levels of IL-6, TNF-α and CRP were significantly lower in the experimental group than in the control group. However, after 7 days of treatment, there were no significant differences in aPTT [26].

A recent randomized clinical trial (RCT) randomized patients to institutional standard prophylactic, intermediate-dose LMWH or unfractionated heparin (100 U/kg subcutaneously twice daily) *vs.* therapeutic dose enoxaparin. The primary efficacy outcome was venous thromboembolism (VTE), arterial thromboembolism (ATE), or death from any cause, and the principal safety outcome was major bleeding at 30 ± 2 days.

Studies found that anticoagulation by unfractionated heparin (UFH) and low-molecular weight heparin (LMWH) is compromised by high affinity binding to circulating histones even in the presence of DNA [27].

aPTT is a routine plasma-based clotting time that is widely used clinically to monitor heparin levels in patients and may be applied to study the neutralization of heparin by histones. The sensitivity of LMWH to histones when FXa-AT complex formation kinetics were studied, which is similar to UFH or LMWH in thrombin-AT systems. It was found that histones could bind both UFH and LMWH with high affinity, relevant to circulating histone concentrations expected during some disease states. Circulating histones are likely to interfere with heparin therapy. Blood clot stabilization by histones is reduced by UFH, so the outcomes are not explained by simple charge interactions [28].

**Fig. 2:**
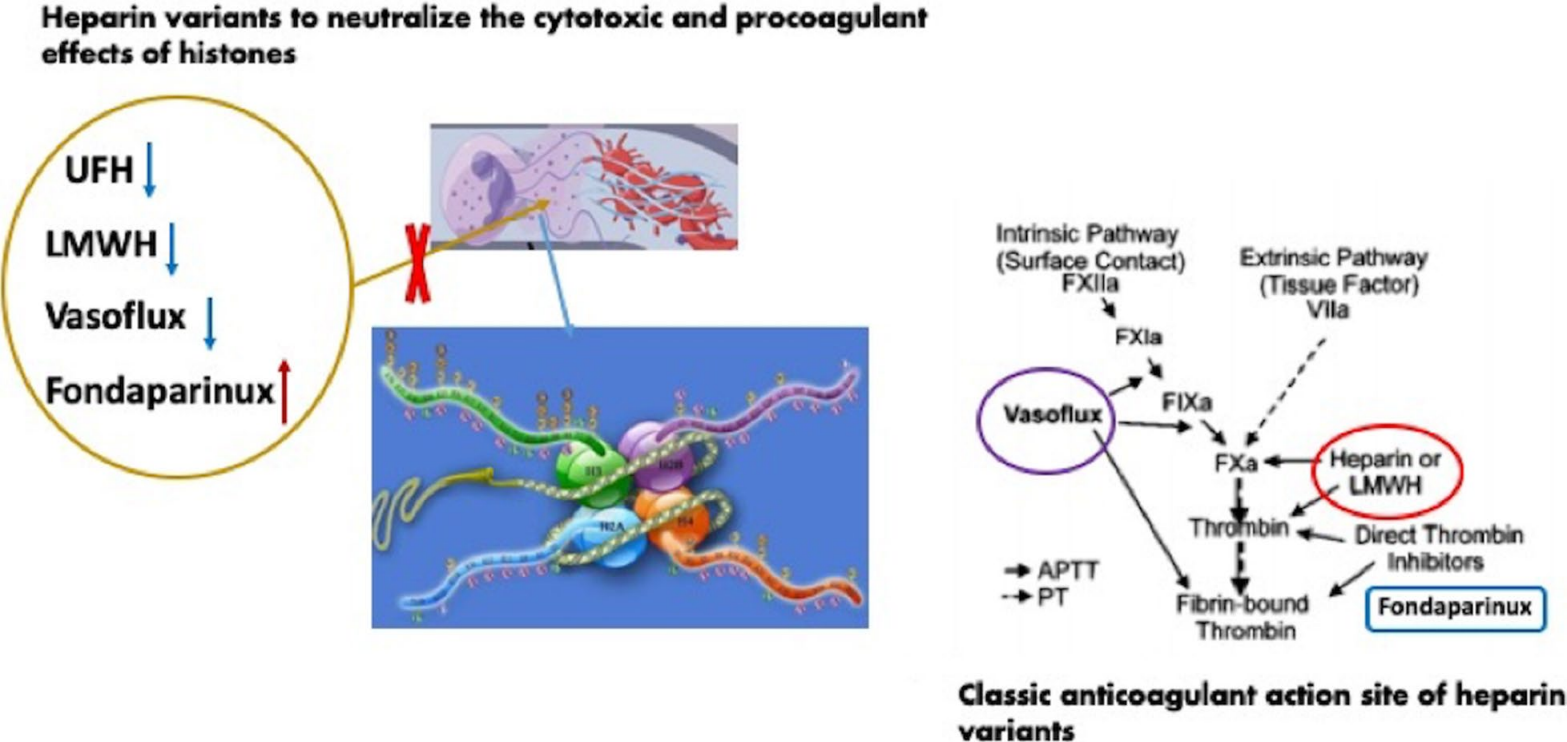
Heparin variants neutralize the cytotoxic and procoagulant effects of histones. Studies illustrated that many heparin variants could neutralize the cytotoxic and procoagulant effects of histones. Compared with UFH, LMWH and vasoflux, fondaparinux are proven to have a greater effect on neutralizing the procoagulant activity of histones

Furthermore, the effectiveness of UFH, LMWH, vasoflux, and fondaparinux in neutralizing the cytotoxic and procoagulant activities of histones was compared. Vasoflux was designed in the 1990s as a new anticoagulant that inactivates fibrin-bound thrombin and blocks FXa generation [29]. The superiority was not confirmed in clinical trials and the molecule was not approved. Nevertheless, this molecule could be repositioned to prevent immunothrombosis. The ability of heparin to fulfill this function requires heparin fragments > 1.7 kDa and is independent of the antithrombin-binding pentasaccharide, suggesting that heparin variants may have differential therapeutic potential in vascular diseases associated with elevated levels of histones (Fig. 2) [30]. There was also uncertainty regarding the effects of combining the LMWH and deoxyribonuclease (DNase I), which can destroy NETs. To address this uncertainty, the combination of DNase I and low-molecular weight heparin (LMWH) was applied in a murine model of abdominal sepsis. Studies illustrated that administration of either DNase I or LMWH could improve the survival of septic mice compared with saline and combination-treated mice. Combination-treated mice showed a small but insignificant improvement in survival compared with saline-treated cecal ligation and puncture mice. Monotherapies improve survival by reducing blood bacterial loads, citrullinated histone-H3, and thrombin-antithrombin complexes and increasing protein C levels [31].

However, Lelliott et al. recently illustrated that heparin (at a concentration of 10 IU/mL), except low molecular weight heparin, fondaparinux, and heparan sulfate, can directly induce NET formation [32].

The formation of NETs is called NETosis. PMA-induced NETosis is “suicidal” but pathogen-induced NETosis is “vital” and “enucleated” neutrophils conserve its ability to perform phagocytosis and antigen presentation. Current studies have shown that inhibition of NETs can dissolve immune thrombosis formed by NETosis, thus mitigating vascular obstruction and promoting functional recovery of patients after stroke. Therefore, inhibiting the formation of NETs and finding the factors that inhibit the formation of NETs are the bottleneck points in solving this key problem. Many studies have illustrated the essential role of citH3 as a NET marker in organ injury. CitH3 induces alI by activating caspase-1 in bone marrow-derived macrophages and bone marrow-derived dendritic cells. CitH3 is an important mediator of inflammation and mortality during sepsis-induced ALI [33].

Tissue factor pathway inhibition by NET-bound serine proteases (NE, cathepsin G) has a prothrombotic effect [34]; previous studies have focused on its participation in the formation of venous thrombosis. However, NETs have been found in both venous and arterial thrombi, and they can serve as scaffolds for platelets and red blood cells, facilitating their adhesion and aggregation [35, 36]. NETs can bind plasma proteins, including fibronectin and von Willebrand factor (vWF), further contributing to thrombus formation [37]. Extracellular DNA and histones, the composition of NETs, influence coagulation. The levels of NET markers correlate with disease severity in thrombotic events, including both thrombotic microangiopathies and deep vein thrombosis [38].

NETosis resulted in neutrophil extracellular traps (NETs) which can contribute to the pathogenesis of endotoxemia and organ damage. NETosis (and NETs) contribute to the pathogenesis of septic shock and organ damage, especially in disseminated intravascular coagulation. Studies have illustrated that PAD-NET-CitH3 can play an important role in pulmonary vascular dysfunction and the pathogenesis of lethal endotoxemia. Many studies, including preclinical and clinical studies, have evaluated the role of PADs and NETs in major trauma, bleeding and traumatic brain injury. NETs formation and PAD activation have been shown to contribute to the postinjury inflammatory state, leading to a detrimental effect on organ systems [39]. Case reports illustrated serial changes of neutrophil extracellular traps in tracheal aspirate of patients with acute respiratory distress syndrome. The formation of NETs was observed in the tracheal aspirate of patients with ARDS. The amount of NETs formation changed dynamically over the clinical course [40]. However, bronchoalveolar lavage fluid from 100 critically ill patients undergoing bronchoscopy for clinically suspected ventilator-associated pneumonia (VAP) was collected. MPO-DNA concentrations were highly correlated with NET formation in the alveolar space. Alveolar concentrations of MPO-DNA were higher in subjects with ARDS and VAP than in those with ARDS alone (p < 0.0001), and higher MPO-DNA was associated with increased odds of VAP [41].

## ARDS and NETosis/immunohaemostasis

In sepsis, the interaction of immune cells with the activation of coagulation has been described as “immunothrombosis” and it refers to the innate intravascular immune response that causes the generation of thrombin and microthrombi [42]. Why do thrombus events increase in ARDS patients? The concept of "immunothrombosis" was proposed to further understand the formation of infection-related thrombosis. ARDS can lead to damage to vascular endothelial cells, the formation of neutrophil extracellular traps (NETs), and immune thrombosis. Therefore, exploring the molecular mechanism of immune thrombosis in ARDS has potential to reduce medical costs as well as the social and economic burden.

Neutrophils do not only engulf pathogens (phagocytosis), but also release their nuclear content, essentially histones and DNA fragments resulting in a net. These NETs support histones and other granule enzymes like myeloperoxidase (MPO) and neutrophil elastase (NE). These fragments are called NETs for neutrophils extracellular traps, and they enable to trap pathogens and blood cells, including platelets. The mechanisms of NETosis are described. The plasma membrane bursts and NETs are released.

**Fig. 3:**
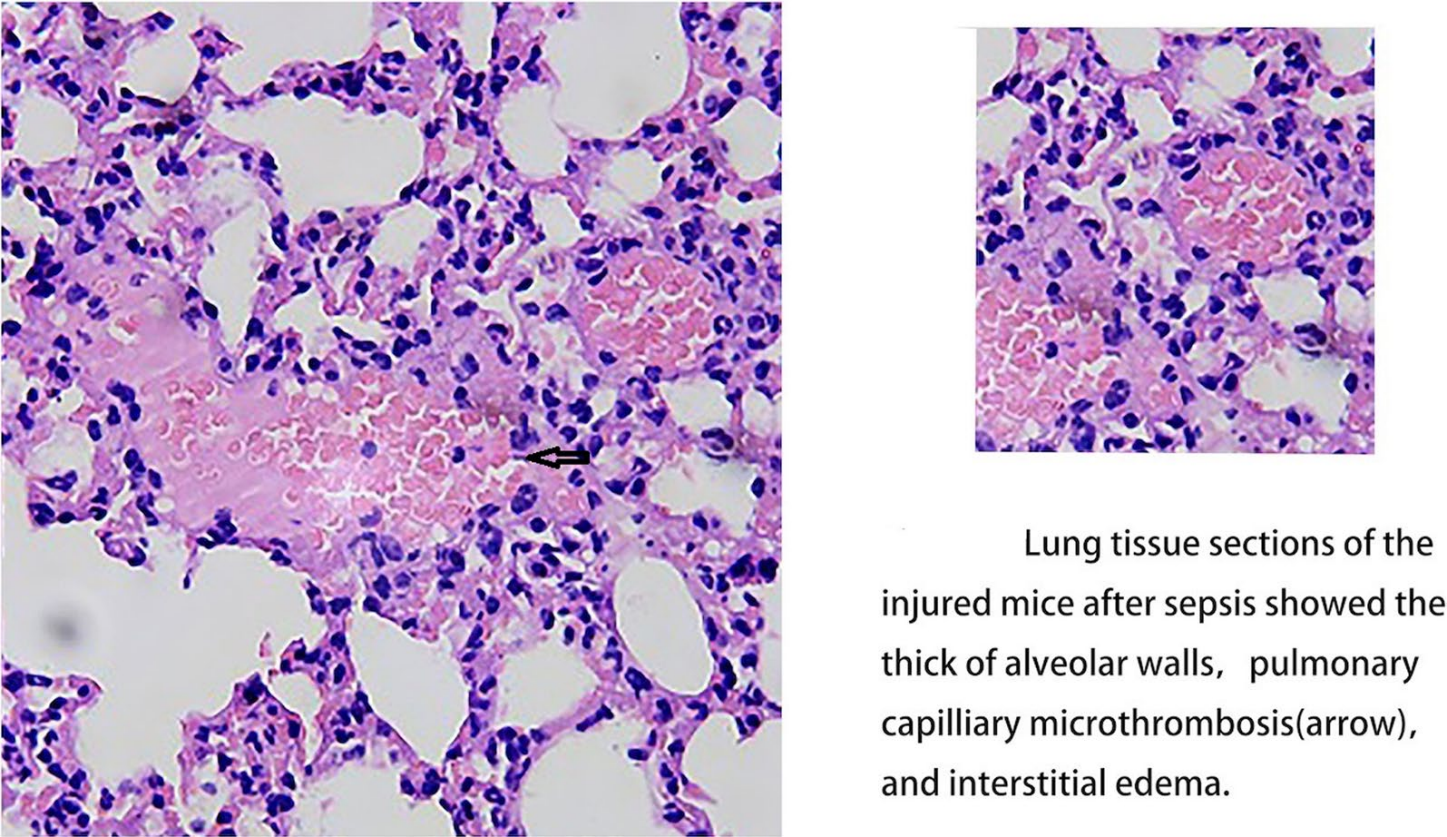
Lung tissue sections of the injured organs after sepsis showed thick alveolar walls, pulmonary capillary microthrombosis, and interstitial edema. The microthrombosis is composed of neutrophil, platelets and erythrocytes

Lung tissue sections from ARDS patients showed thick alveolar walls, pulmonary capillary microthrombosis, and interstitial edema [43], as illustrated in our study (Fig. 3). However, how do microthrombi form in ARDS? Under normal conditions, pulmonary vascular endothelial cells can regulate inflammation and coagulation processes well. The activation of pulmonary vascular endothelial cells in ARDS can cause an imbalance in the regulation of inflammation and coagulation. Tissue Factor (TF) is also involved, triggering the cascade of exogenous coagulation activation and causing thrombosis.

Neutrophils release NETs via a multistep process called NETosis. Neutrophils release NETs in host defense are suicidal NETosis and vital NETosis [44]. Suicidal NETosis involves the triggering of nicotinamide adenine dinucleotide phosphate (NADPH) oxidase (NOX) dependent pathways [45]. Briefly, the activation of receptors increases calcium ions and stimulates the activity of protein kinase C (PKC) and NOX, leading to the formation of ROS. Activation of MPO participates in the depolymerization of chromatin and the rupture of the nuclear membrane, forming the main components of NETs. It also activates NE, promoting the transfer of NE from the cytoplasm to the nucleus, cleaving chromatin and releasing it into the cytoplasm. In addition, MPO also contributes to the decondensation of chromatin. In this process, the peptidyl arginine deiminase 4 (PAD4) enzyme is activated [46]. The combined action of PAD4, NE, and MPO results in citrullination of histone H3 and subsequent chromatin decondensation, discharging into the extracellular space and resulting in neutrophil death. This process is also called NOX dependent NETosis [47].

Citrullinated histone H3 (citH3) produced in neutrophils is a specific component of NETs. Many recent studies have shown that citH3 is associated with the severity of acute lung injury and other diseases and can predict the clinical prognosis of these patients [33, 48]. Recent studies have also shown that the exposition of citH3 is increased in acute lung injury after infection [49]. Severe traumatic brain injury (sTBI) induced ALI and caused an increase in the wet/dry weight ratio and lung vascular permeability, and sTBI promoted the oxidative stress response in the lung. sTBI promoted NET formation, mainly increasing the decorating of MPO, NE and CitH3 in addition to the release of inflammatory cytokines, such as IL-6, IL-1β, TNF-α and MCP-1 [50]. Further studies found that PAD2 and PAD4 participate in the digestive system, from inflammatory to oncological diseases, providing related therapeutic prospects [51].

**Fig. 4:**
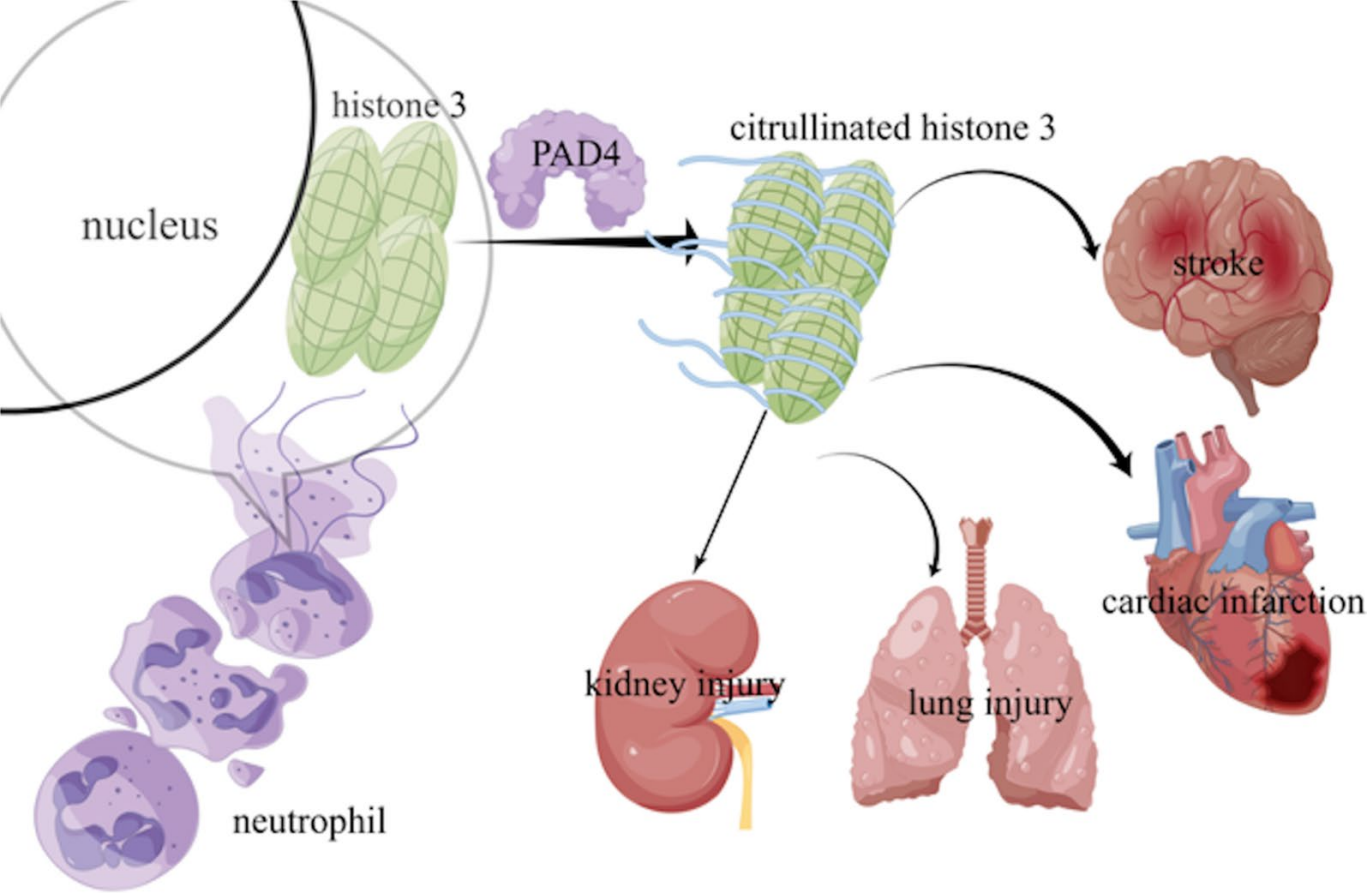
PAD4 is involved in the formation of NETs and is the key enzyme that posttranslationally modifies histones into conciliated histones (citH3). citH3 was reported to participate in the process of multiple organ injury, including stroke, lung injury, kidney injury, and cardiac infarction

In addition to toxic effects on human endothelial and other cells in vitro, injection of histone in mice resulted in microthrombus formation, organ failure, and death. The process of NETs formation requires the posttranslational modification of histones into citrullinated histones (citH3). This process relies on the catalysis of peptide acylarginine deiminase (PAD) [52], in which PAD4 is involved in the formation of NETs and is a key enzyme that catalyzes the conversion of arginine to citrulline (Fig. 4). When neutrophils are stimulated, they must change from PAD4-mediated histone deamination to citrullinated histones citH3 and citH4, allowing their chromatin to untangle, followed by changes in nucleus morphology and nuclear membrane rupture to form NETs. This is a key step in NET formation [53].

Sepsis-related deaths accounted for approximately 20% of all global deaths in 2017 [54]. Many studies have demonstrated that NETs contribute to disease severity in sepsis [55, 56]. CitH3 was reported to participate in the process of disease severity [57]. The levels of citH3 were elevated significantly in septic acute pancreatitis (AP) patients compared with those in nonseptic AP patients and healthy volunteers. Furthermore, citH3 concentration was positively correlated with PAD2, PAD4, dsDNA concentration, and Sequential Organ Failure Assessment scores. CitH3 concentration was increased in septic AP patients and was closely correlated with disease severity and clinical outcomes, which may potentially be a diagnostic and prognostic biomarker of septic AP [58]. Clinical studies illustrated that levels of circulating citH3 at enrollment were significantly increased in septic shock patients compared to patients hospitalized with noninfectious shock [59]. Inflammation or plasma inflammatory mediators such as S100A8/A9 or NETosis markers such as histone/DNA complexes and cfDNA may play a role in the pathogenesis of iTTP, which may help stratify patients with a high risk of death during acute immune-mediated thrombotic thrombocytopenic purpura (iTTP) episodes [60]. Citrullinated histone H3 (CitH3), as a NET component, is elevated in both septic animals and patients, and the presence of citH3 is a reliable diagnostic biomarker of sepsis. A standardized mouse model of cecal ligation and puncture (CLP)-induced sepsis established a reliable blood biomarker of sepsis [61]. Blocking circulating citH3 might be a potential therapeutic method for the treatment of endotoxemia [62].

Citrullination of nuclear histones by PAD4 can lead to decondensation of chromatin, which is a key step in the onset of NET formation. NETs are formed within the vasculature during sepsis in response to circulating pathogens [63]. Patients with pneumosepsis, compared with those with no pulmonary disease, show increased citrullinated histone 3 (citH3) levels in their airways and a trend toward elevated levels of NET markers, including cell-free DNA and nucleosomes. NETs formation and host defense were studied during pneumonia-derived sepsis induced by *Klebsiella pneumoniæ* in PAD4^+/+^ and PAD4^−/−^ mice. CitH3 levels were increased in the lungs of PAD4^+/+^ but not PAD4^−/−^ mice [64]. The involvement of PAD4 in several diseases signifies the need for a PAD4 inhibitor. Although progress has been made to produce an isotype specific and potent PAD4 inhibitor, no PAD4 inhibitor is currently ready for clinical use [65]. GPR109A belongs to the G protein-coupled receptor family [53]. Recently, some studies have shown that GPR109A plays an important role in inflammatory diseases such as sepsis and nephrosis [66, 67]. The regulatory effect of GPR109A on early NETs formation was identified for the first time and provides a new target for the treatment of sepsis. Mechanisms can involve the production of ROS, which can inhibit PAD4 activity [68].

Extracellular DNA leads to thrombin generation in patients with sepsis. Extracellular histones released from NETs increase plasma thrombin generation through TLR2 and TLR4 by activating platelets [69]. Inflammation-driven activation of neutrophils, platelets, and vascular endothelial cells may promote a state of immune thrombosis, which is most likely driving disease severity in COVID-19 patients [70].

Studies have demonstrated that treatment with forsythiaside B (FTB) inhibits NET formation and downregulates PAD4 expression in peripheral neutrophils. The effects of FTB on coagulopathies were similar to those of monotherapy with NETs or PAD4 inhibitors, which can alleviate coagulopathies in rats with sepsis. The underlying mechanism of the effect of FTB consists of the inhibition of PAD4-dependent NET formation [71].

NET-Inhibitory Factor (nNIF), an endogenous NET inhibitor, Cl-amidine, a PAD4 inhibitor, and DNase I, a NET-degrading enzyme, could decrease peritoneal NET formation and inflammatory cytokine levels at 24 h compared to controls. Finally, nNIF administration significantly improved survival in mice treated with suboptimal doses of meropenem even when treatment was delayed until 2 h after peritonitis induction [72].

Phenotypic data from primary immune cells provide the first evidence that PAD4-selective small-molecule inhibitors can affect cellular citrullination and mimic the deficiency in NETs production previously reported in PAD4-deficient mice [73]. PAD4 mediates the citrullination of serine protease inhibitors or SERPIN (antithrombin, C1-inhibitor), fibrinogen and the vWF-cleaving protease ADAMTS13 [74].

Citrullination of serine protease inhibitors can result in activity loss, resulting in dysregulation of blood coagulation [54]. Citrullination of antithrombin abolished its ability to inhibit thrombin activation, and increased levels of citrullinated antithrombin in the plasma of RA and colorectal adenocarcinoma patients did not show an association with thrombosis [75]. Additionally, PAD4 injection in vivo can induce citrullination of plasma proteins, thereby reducing the ability of ADAMTS13 to cleave VWF, resulting in the formation of prothrombotic vWF-platelet strings [76].

## Therapeutic opportunities: targeting PAD4 via PAD4 inhibitors and lncRNAs.

Studies have shown that inhibition of PAD4 activity can inhibit the formation of NETs in mice and humans (Table 1) [77]. Previous studies have shown that calcium ions, in vivo redox state and sodium bicarbonate concentration can affect the activity of PAD4 [78]. Calcium ionophores, such as ionomycin and A23187, are therefore frequently used to activate PAD4 in vitro [79]. Calcium is known as a key regulator of PAD4 catalytic activity and has the capacity to induce structural changes in PAD4, which can accompany the transition of an inactive to an active PAD4 conformation [80–82].

**Table 1:** Intervention of PAD4 as therapeutic target biomarkers for NETs activation

Reference	Inhibitor	Study object	Action site	Regulation
Guo W et al (2023) [68]	GPR109A	Mice and human neutrophil	ROS	
Molinaro R et al (2022) [78]	GSK484	Mice	PAD4	
Liu Y et al (2017) [81]	Ionomycin	Digital model	Ca2 +	
Liu Y et al (2017) [81]	A23187	Digital model	Ca2 +	
Zhao X et al (2021) [89]	TDFA	Mice	NF-κB	
Qian O (2020) [82]	TcpC	Mice and human neutrophil	ubiquitination	
Zhou Y et al (2018) [87]	Bicarbonate	Human neutrophil	pH	
Deng H et al (2022) [84]	JBI-589	Tumor mice	CXCR2	
Kolarz B et al (2021) [83]	DAS28	RA patients	methylation	
Hawez A et al (2019) [92]	miR-155	Human neutrophil	promoter	
He, et al (2022) [71]	FTB	Rat and human neutrophil	TLR4/NF-κB	
Gajendran C et al (2023) [85]	JBI-589	Mice	cytochrome P450s	
Denorme et al (2023) [74]	Cl-amidine	Mice	CXCL1	
Lewis et al (2015) [53]	GSK199	Digital model	citrullination	

A recent study also illustrated that rTcpC treatment caused significantly enhanced ubiquitination of PAD4 in neutrophils. The PAD4 gene methylation and anti-PAD4, disease activity score (DAS28) and ACPA level have been illustrated. Elevated methylation is associated with lower disease activity and lower levels of ACPAs and PAD4. The methylation degree in this area increases during effective treatment and might play a role in rheumatoid arthritis (RA) pathophysiology and could be a future therapeutic target [83].

The reduced *PADI4* methylation in RA patients results in increased expression of the PAD enzyme (expression and activity not measured) and, consequently, increased protein citrullination and excessive ACPA production.

A novel selective inhibitor, JBI-589, was illustrated to target PAD4-mediated neutrophil migration to suppress tumor progression. In tumor-bearing mice, pharmacologic inhibition of PAD4 with the selective small molecule inhibitor JBI-589 can result in reduced CXCR2 expression and block neutrophil chemotaxis [84]. JBI-589 would be beneficial for both PAD4 and NET-associated pathological conditions [85].

CXCR1 and CXCR2 agonists proved to be the major mediators of cancer promoted NETosis. Tumor-secreted CXCR1 and CXCR2 ligands induce extrusion of NETs. Tumor cells protected from cytotoxicity by NETs underlie successful cancer metastases in mice and the immunotherapeutic synergy of protein arginine deiminase 4 (PAD4) inhibitors, including GSK484 [86]. The inhibitors of NETosis (JBI-589 and GSK484), currently under development to suppress tumor progression, could also be of interest in ARDS with the rationale.

Inhibitors of PAD4 include GSK199 and GSK484, and NET formation can be inhibited by systematic targeting of nanoparticle drug delivery. GSK484 can inhibit the formation of NETs and mitigate the complications of atherosclerotic thrombosis and involvement in intima lesions [87]^.^ In lung ischemia reperfusion injury, inhibition of PAD4 can prevent remote lung injury caused by renal ischemia reperfusion injury [88]. However, there has been no report on the protective effect against ARDS. Our preliminary experimental results confirmed that the mRNA expression level of PAD4 was increased in the lung tissues of infected mice.

Another PAD4 selective inhibitor is Thr-Asp-F-amidine (TDFA), which can efficiently decrease the severity of lung edema, attenuate the severity of pulmonary injury and improve the survival rate following lethal LPS administration. TDFA also reduced activated cell infiltration and suppressed inflammation-related parameters, including proinflammatory cytokine production (TNF-α, IL-6 and IL-1β) and oxidative stress (MDA, GSH and SOD). Furthermore, TDFA reversed the TEER downregulation tendency and tight junction protein (ZO-1, Occludin, Claudin-4) levels that represent the integrity of the alveolar epithelium. Eventually, TDFA exerts its protective roles by modulating the nuclear localization of the transcription factor NF-κB P65 in epithelial cells, indicating that PAD4 inhibition may serve as a promising therapeutic approach for LPS-induced ALI [89].

Thrombin generation in TFPI-deficient plasma demonstrated reduced anticoagulant activity of citrullinated TFPI. Mass spectrometry demonstrated citrullination of surface-exposed arginine residues in TFPIα after incubation with PAD4 [90].

Non-coding RNAs (ncRNAs) are divided in microRNAs (miRNAs), long ncRNAs (LncRNAs) and circularRNAs (circRNAs). MicroRNAs (miRNAs) are small noncoding RNA molecules that regulate gene expression by binding to messenger RNA. Recent studies have shown that miRNAs are present in exosomes in circulating blood [91]. Exosomes are nanosized membranous vesicles (30–150 nm) that can be secreted by a variety of cells, mainly epithelial cells, tumor cells and macrophages. Exosomes contain lipids, proteins, messenger RNA (mRNA), and miRNAs involved in intercellular interactions. miRNA in exosomes has become an important regulatory factor for cell function. Previous studies have shown that intramuscular injection of hypoxic exosomes after infarction can improve the left ventricular function of the heart and reduce the infarct volume, and exosomes derived from hypoxic bone marrow mesenchymal stem cells are rich in miR-125b-5p [92]. miR-155 is located in the third exon of the B-cell Integration Cluster (bic) gene on the human chromosome and is one of the most important miRNAs in the immune system. miR-155 is released into the blood by exosomes and plays a variety of functions in the immune system, accompanied by a very important role in inflammation. Recent studies have shown that miR-155 activates the mRNA of PAD4 after exogenous stimulation of neutrophils [93]. Our previous review also showed that exosomal miR-155 plays an important role in chronic low-grade inflammation caused by obesity [94]. Studies have confirmed the increased expression of exosomal miR-155 in macrophages during acute lung injury caused by sepsis [95], miR-155 can regulate the formation of NETs in the lung tissue of septic mice [96], and miR-155 regulates the expression of PAD4 and NETs during exogenous neutrophil stimulation. However, it has not been reported whether miR-155/PAD4 is involved in the formation of NETs during immune thrombosis in ARDS patients.

Rinn et al. illustrated that lncRNAs are involved in various biological processes, including genomic imprinting, embryonic development, cell differentiation, immune modulation, tumor metastasis, and cell cycle regulation, which are also implicated in innate immune responses, as evidenced by various TLR engagements [97–99]. Carpenter et al. found that a large intergenic noncoding RNA (lincRNA), lincRNA-Cox2, could mediate both the activation and regression of certain classes of immune-related genes [100]. LINC00324 positively upregulated PAD4 expression by interacting with miR-3164 and recruiting HuR protein. The LINC00324/miR-3164/PAD4 axis modulated the PI3K/AKT pathway in nasopharyngeal carcinoma cells (NPC). Moreover, the upregulation of PAD4 counteracted the influences of LINC00324 deficiency on NPC cell proliferation, apoptosis, and autophagy and on NPC tumor growth in mice [101]. LncRNA OIP5-AS1 was upregulated and miR-223 was downregulated in the serum of ARDS patients and LPS-treated HPMECs. LncRNA OIP5-AS1 aggravates LPS-induced ARDS via the miR-223/NLRP3 axis and provides new targets for ARDS therapy [102]. However, whether it affects the mechanism of PAD4 requires further study.

## Conclusion

Lung is the first easily damaged organ with sepsis. Neutrophils release NETs via a multistep process called NETosis. Activation of MPO participates in the depolymerization of chromatin and the rupture of the nuclear membrane, forming the main components of NETs. MPO also contributes to the decondensation of chromatin. PAD4 enzyme is activated. The combined action of PAD4, NE, and MPO results in citrullination of histone H3 and subsequent chromatin decondensation. The activation of NETs in ARDS can cause an imbalance in the regulation of inflammation and coagulation. PAD4 is the key enzyme that regulates the NETs skeleton protein histone H3 to citrulline histone in NETs. Inhibitors of PAD4 such as Calcium, rTcpC, JBI-589, GSK199, GSK484 and TDFA could be therapeutic target biomarkers for NETs activation.

## Data Availability

Not applicable.
